# Supplementary material for: DNA Methyltransferase 3 (MET3) is regulated by Polycomb group complex during Arabidopsis endosperm development
Source: Plant Reprod. 2022 Jan 28;35(2):141–51. doi: 10.1007/s00497-021-00436-x (PMC9110472; doi:10.1007/s00497-021-00436-x)
Supplement: Supplementary file 1 — Supplementary file1 (PDF 34739 kb) [file 497_2021_436_MOESM1_ESM.pdf]

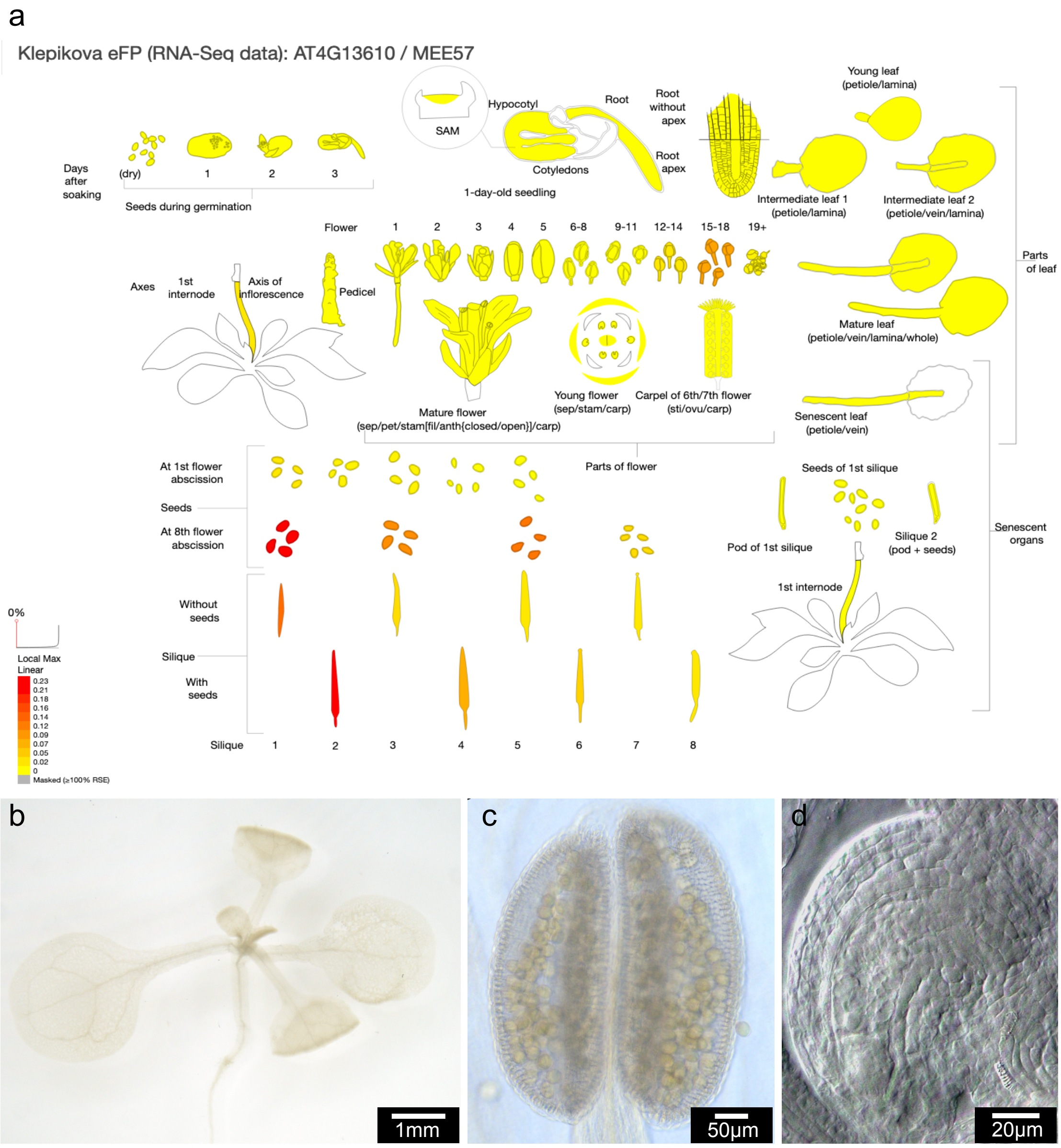


Figure S1. *MET3* is only expressed in seeds

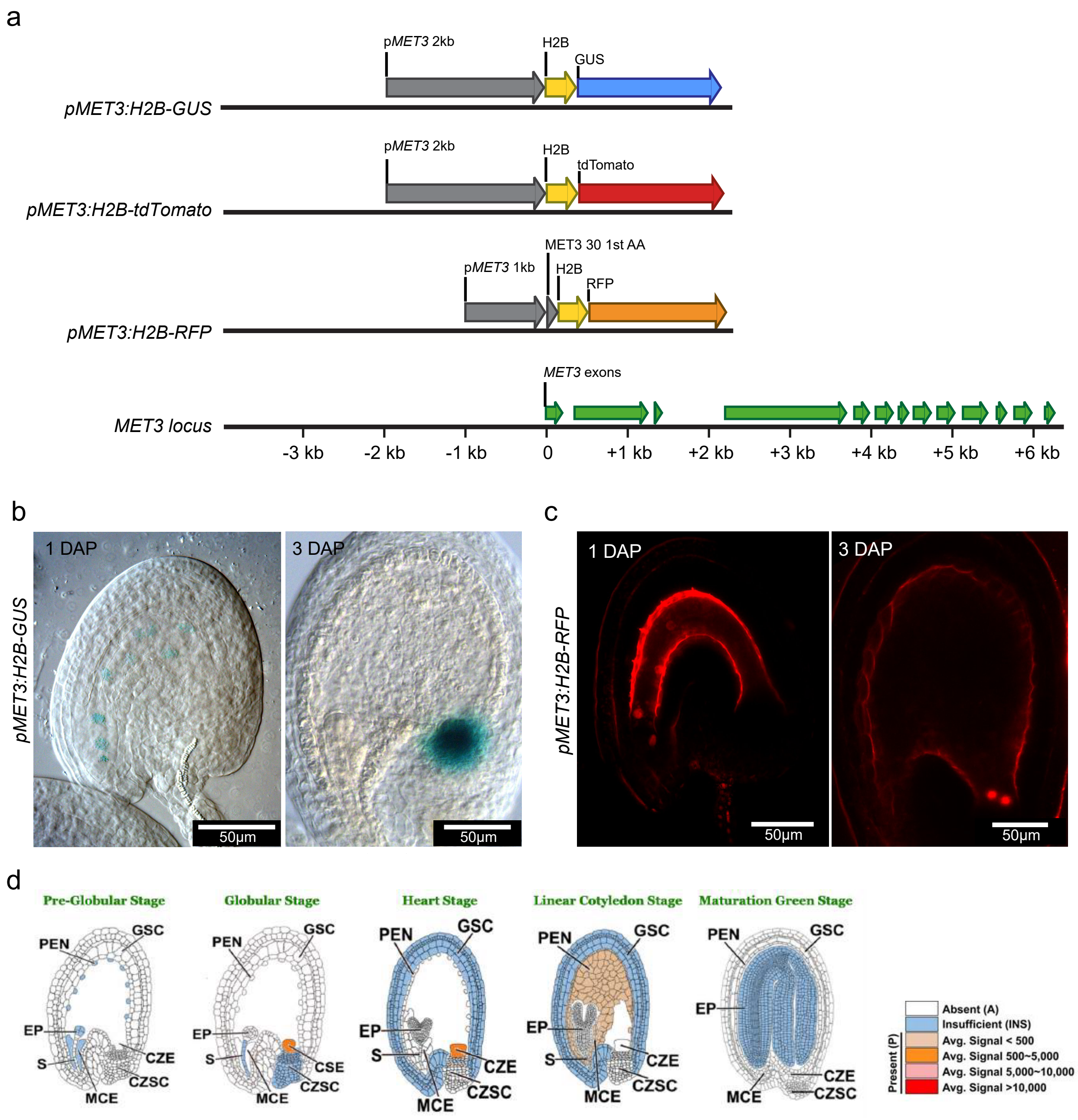


Figure S2. *MET3* reporters and expression

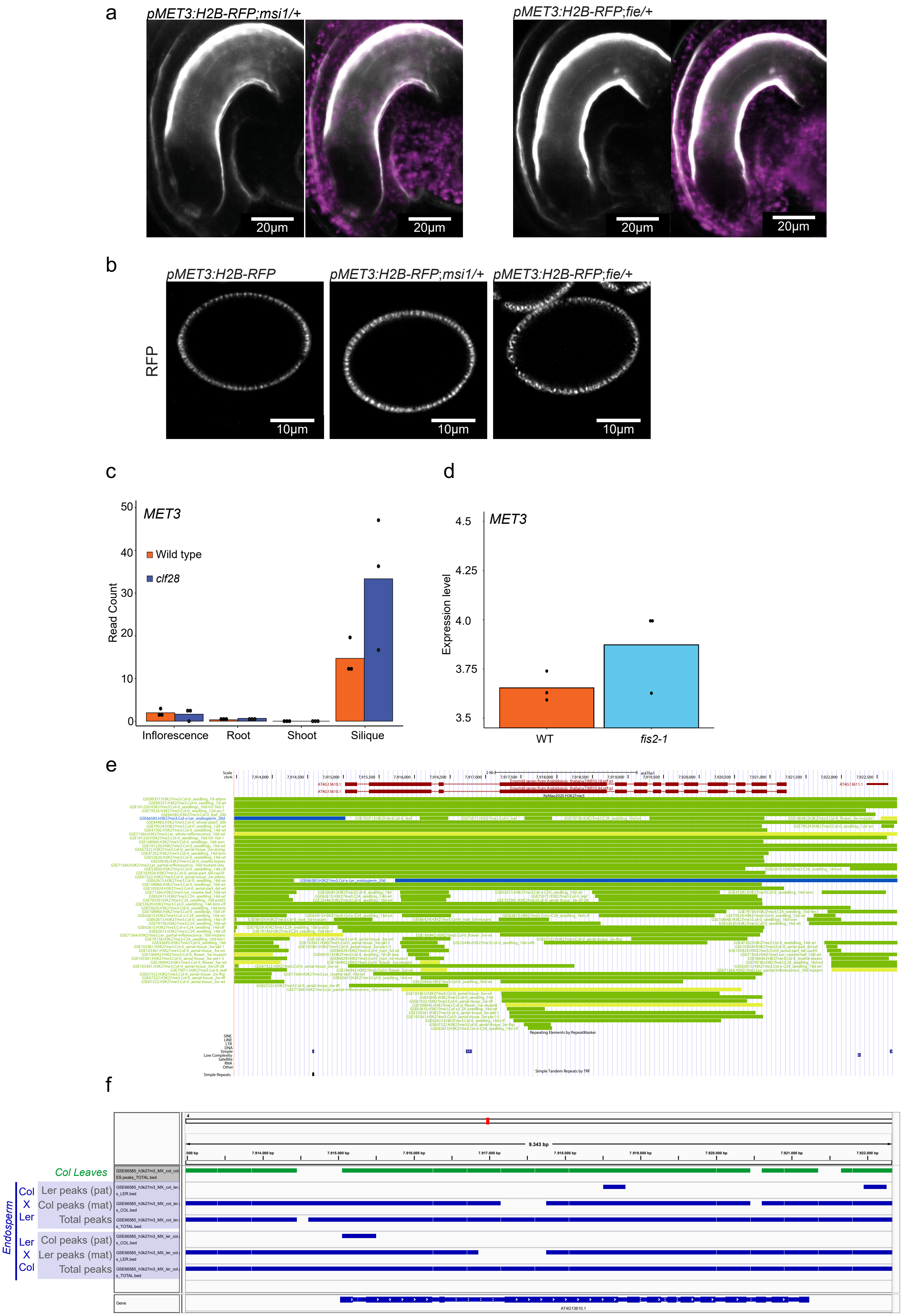


Figure S3. MET3 regulation by Polycomb group

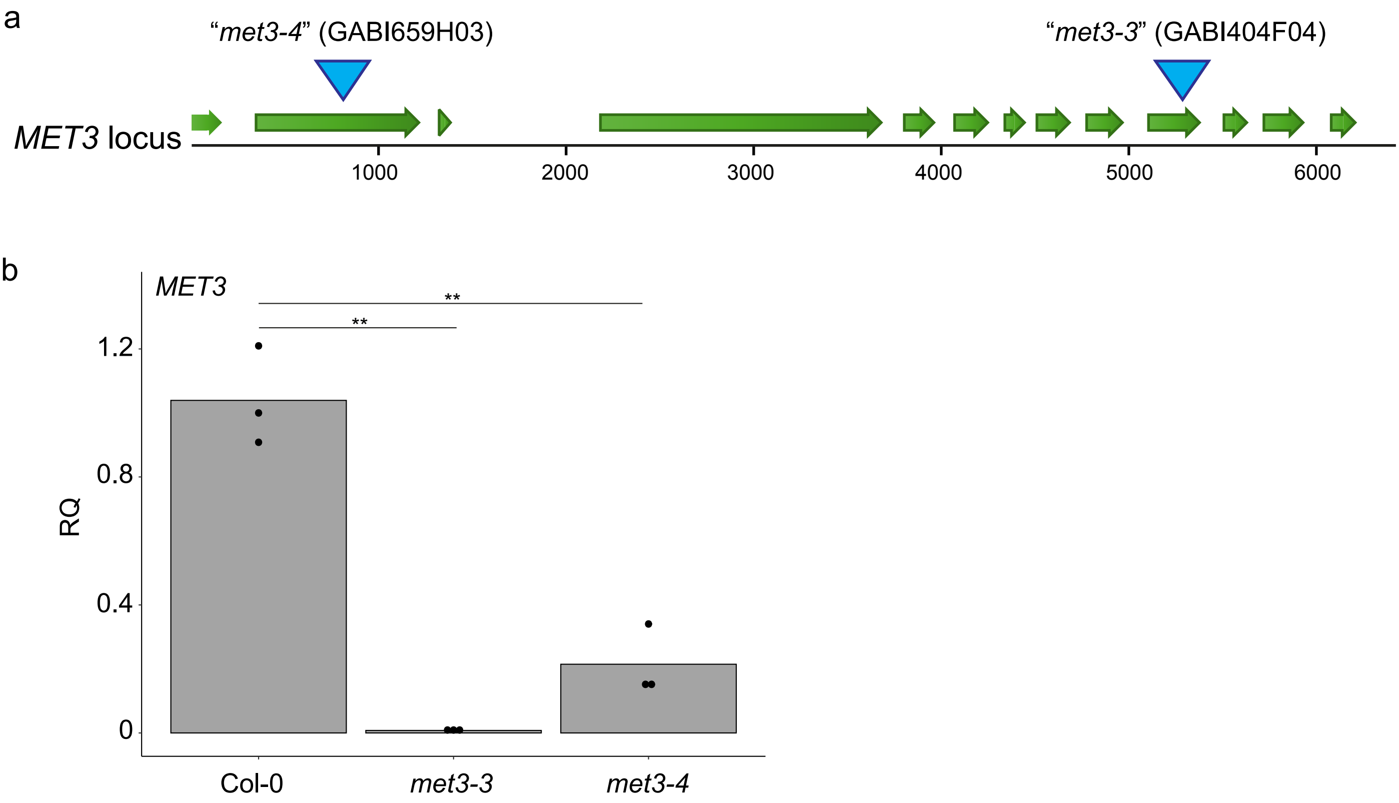


Figure S4. Characterization of *MET3* mutants

a

**Transmission rate**

| Progeny genotype | +/+ | -/+ | -/- | Total | Chi Square score |
|---|---|---|---|---|---|
| *met3-3* | 44 (25%) | 97 (55.11%) | 35 (19.88%) | 176 (100%) | 0.207 |
| *met3-4* | 39 (23.07%) | 85 (50.09%) | 45 (26.62%) | 169 (100%) | 0.806 |

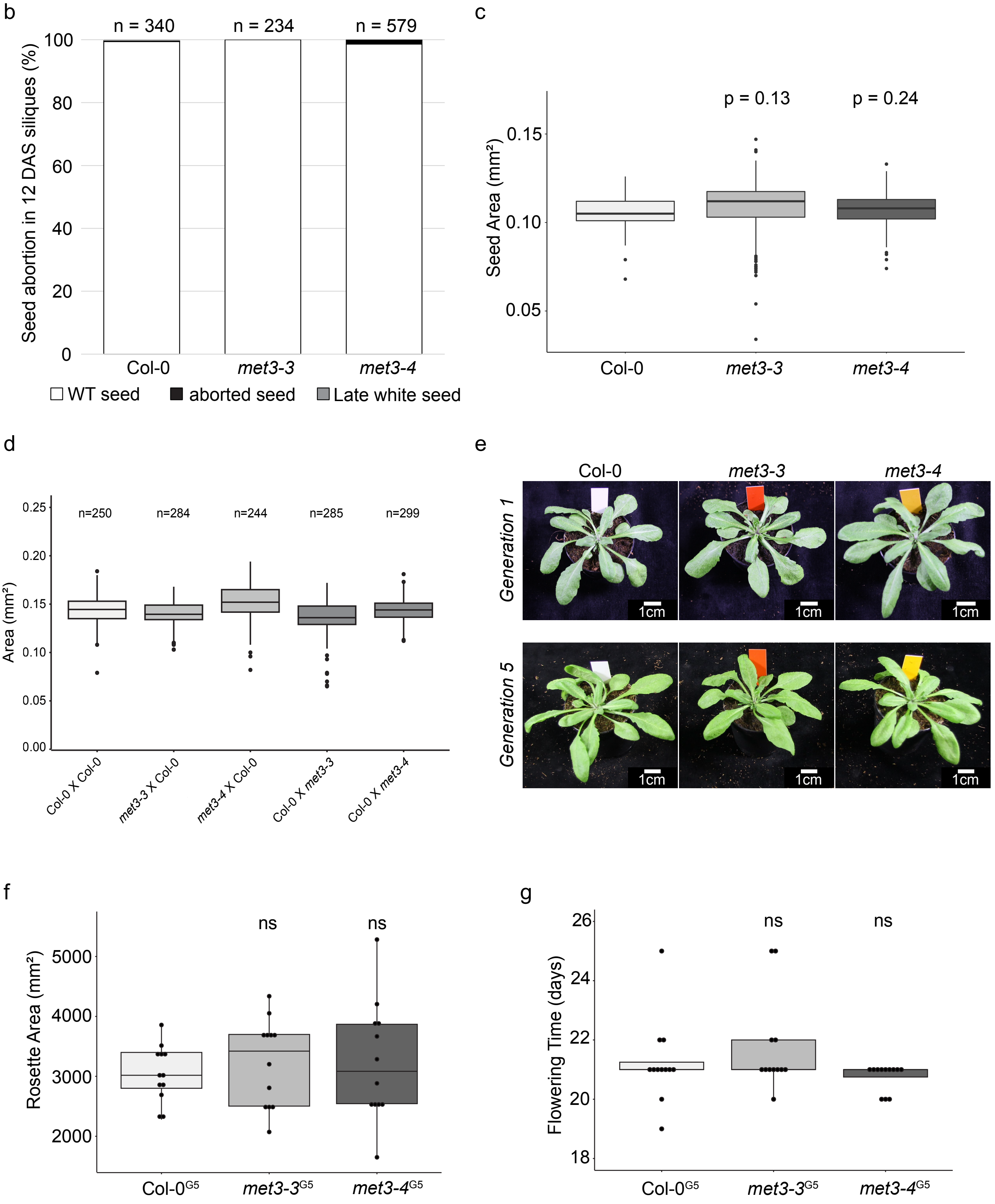


Figure S5. MET3 mutants do not show developmental defect

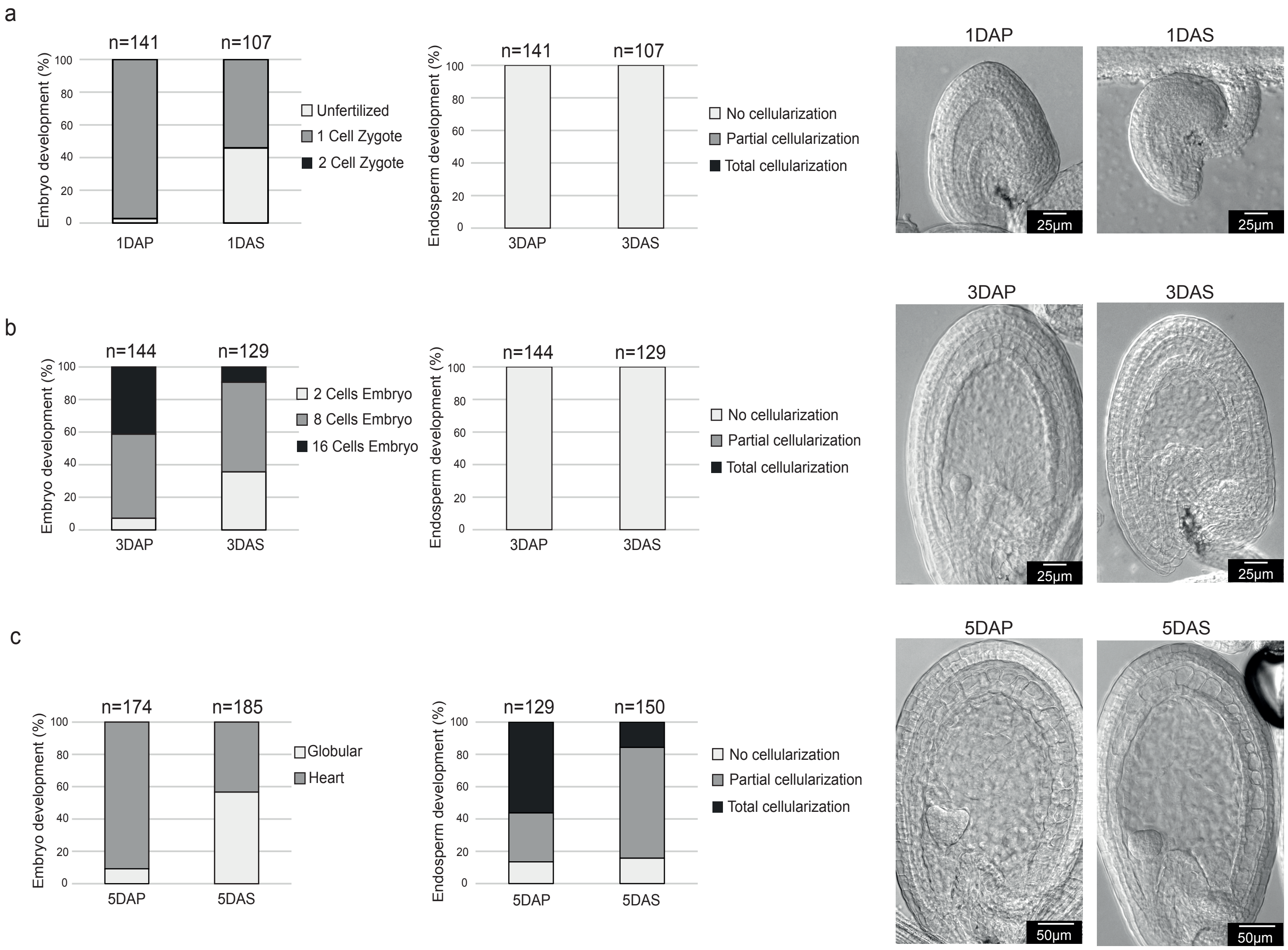


Figure S6. Side-by-side comparison between Day After Pollination (DAP) and Day After Synchronization (DAS).

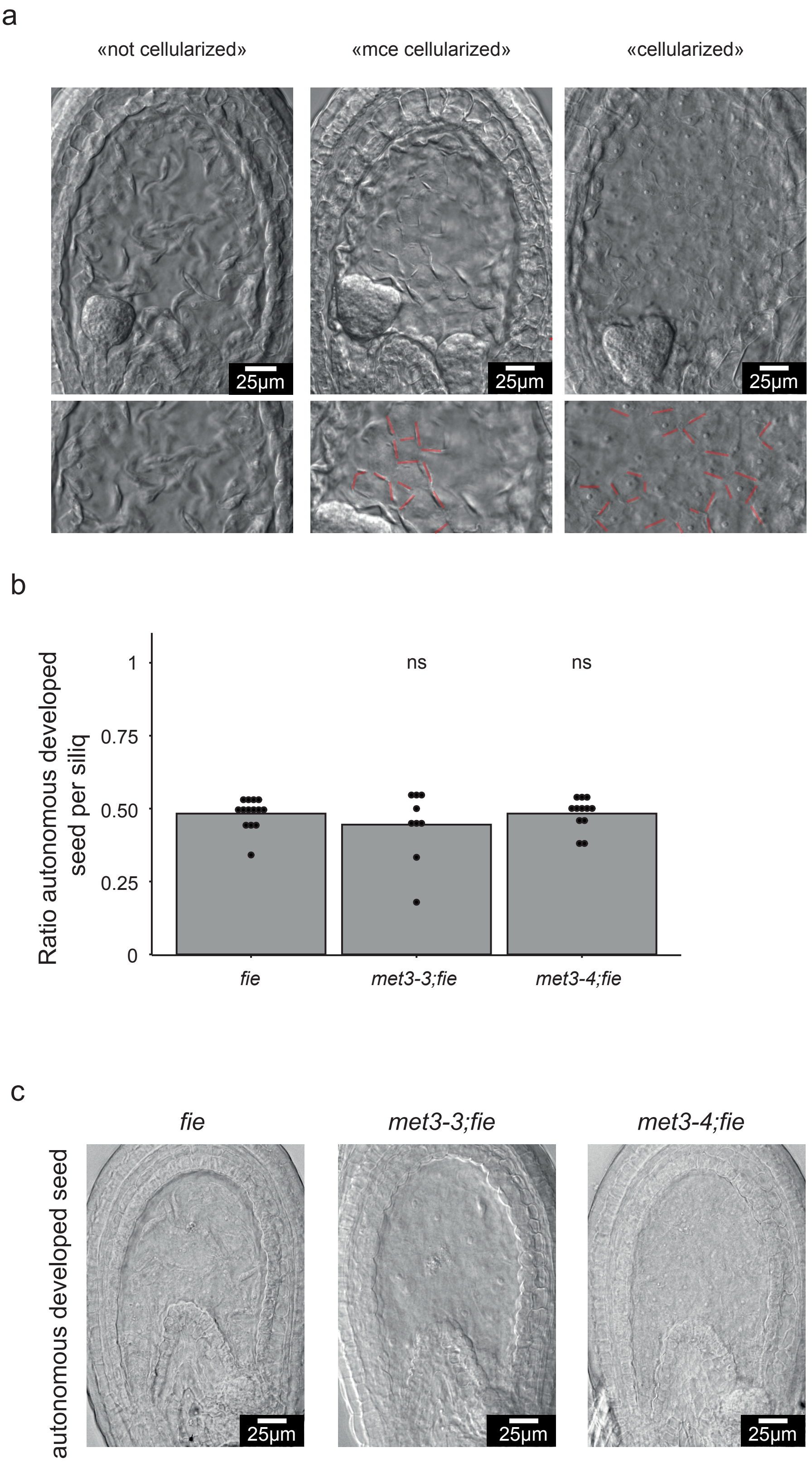


Figure S7. MET3 does not influence fie phenotype

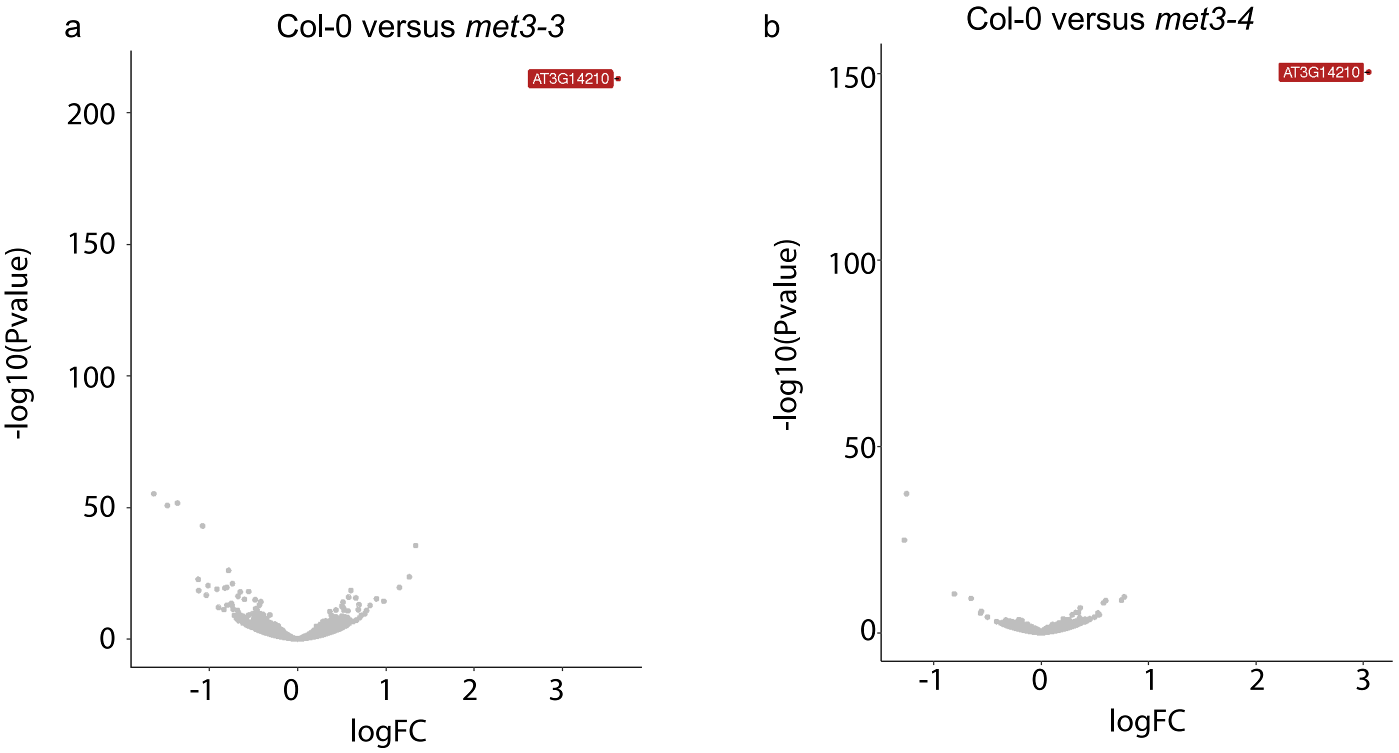


Figure S8. Transcriptome of *met3* mutant seeds at 3DAP
